# Multitarget Molecular Hybrids of Cinnamic Acids

**DOI:** 10.3390/molecules191220197

**Published:** 2014-12-02

**Authors:** Aikaterini Peperidou, Dorothea Kapoukranidou, Christos Kontogiorgis, Dimitra Hadjipavlou-Litina

**Affiliations:** 1 Department of Pharmaceutical Chemistry, School of Pharmacy, Faculty of Health Sciences, Aristotle University of Thessaloniki, Thessaloniki 54124, Greece; E-Mail: peperidou@pharm.auth.gr; 2 Department of Physiology, School of Medicine, Faculty of Health Sciences, Aristotle University of Thessaloniki, Thessaloniki 54124, Greece; E-Mail: dkapoukr@auth.gr; 3 Laboratory of Hygiene and Environmental Protection, Medical School, Democritus University of Thrace, Dragana (Campus), Alexandroupolis 68100, Greece; E-Mail: kontog@pharm.auth.gr

**Keywords:** hybrids, cinnamic acids, multitarget, lipoxygenase inhibitors, antiproteolytic activity, analgesics, anti-inflammatories

## Abstract

In an attempt to synthesize potential new multitarget agents, 11 novel hybrids incorporating cinnamic acids and paracetamol, 4-/7-hydroxycoumarin, benzocaine, *p*-aminophenol and *m*-aminophenol were synthesized. Three hybrids—**2e**, **2a**, **2g**—and **3b** were found to be multifunctional agents. The hybrid **2e** derived from the phenoxyphenyl cinnamic acid and *m*-acetamidophenol showed the highest lipoxygenase (LOX) inhibition and analgesic activity (IC_50_ = 0.34 μM and 98.1%, whereas the hybrid **3b** of bromobenzyloxycinnamic acid and hymechromone exhibited simultaneously good LOX inhibitory activity (IC_50_ = 50 μM) and the highest anti-proteolytic activity (IC_50_= 5 μM). The hybrid **2a** of phenyloxyphenyl acid with paracetamol showed a high analgesic activity (91%) and appears to be a promising agent for treating peripheral nerve injuries. Hybrid **2g** which has an ester and an amide bond presents an interesting combination of anti-LOX and anti-proteolytic activity. The esters were found very potent and especially those derived from paracetamol and *m*-acetamidophenol. The amides follow. Based on 2D-structure–activity relationships it was observed that both steric and electronic parameters play major roles in the activity of these compounds. Molecular docking studies point to the fact that allosteric interactions might govern the LOX-inhibitor binding.

## 1. Introduction

In recent years, intensive research has been conducted on cinnamic acid derivatives, seeking to create new polyfunctional drugs based on them [1,2]. This class of compounds is of additional interest because some representatives cinnamic acids such as caffeic, ferulic acid, *etc.* are widely represented in plants and together with flavonoids, participate in the regulation of many biochemical processes. The increased interest in this group of compounds is due to the variety of their pharmacological activities: e.g., antioxidative [3], antitumor [4], and antimicrobial [5], as well as to their ability to regulate the activity of a number of enzyme systems that participate in cell activation processes. Simple cinnamic acids, for example caffeic and ferulic acids, inhibit 5- and 12-1ipoxygenases [6], reduce the level of leukotrienes in tissues, and may potentially be used to treat bronchial asthma [7].

Usually the drugs commonly used in therapy have been developed on the basis of the “one target” approach and they are successfully used in single-target therapy. For the treatment of complex diseases, in which more than one target is implicated, a combination of drugs is used. In this regard, nowadays there is a great interest in the use of hybrid multi-target drugs. Hybrid drugs combine two drug entities in a single molecule, a new chemical entity more medically effective than its individual components and capable of interacting simultaneously with multiple targets, directly or following metabolism [8,9].

In the last decade we have designed and synthesized [10,11,12,13,14] several cinnamic acids as potent lipoxygenase inhibitors, antioxidants and anti-inflammatories. As a tool in our design we used our published QSAR results [15]. Considering also the biological role of coumarin derivatives on the previous mentioned targets, we found it interesting to combine these particular molecules in our molecular hybridization. Since serine proteases are implicated in the pathophysiology of inflammatory diseases we have included trypsine, a serine protease, in our multitarget design.

In this light molecules were designed by molecular hybridization of three drugs: (i) hymechromone (4-methyl-7-OH-coumarin); (ii) paracetamol; (iii) benzocaine, as well as of some drug-like molecules: (i) 7-OH-coumarin; (ii) 4-OH-coumarin; (iii) *p*-aminophenol; (iv) *m*-aminophenol; (v) *m*-aminoacetamide and cinnamic acids.

The design principle was aimed at combining the synergistic property of our previously synthesized potent cinnamic acids with the sequestering activity of these drugs/drug-like molecules to get new cinnamic acid hybrids that might act as effective multitarget bioactive agents. Yet another objective of the study was to evaluate the effect of steric and electronic parameters on anti-lipoxygenase activity and to optimize the activity through systematic modification of the substituents on the phenylacrylamide core.

## 2. Results and Discussion

### 2.1. Chemistry

**Figure 1:**
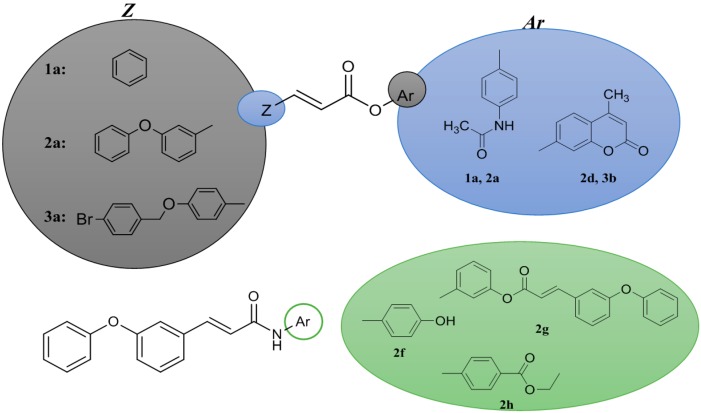
Cinnamic acid-based hybrids.

To design new cinnamic acid-based drug candidates, we divided the projected molecules into three parts: the cinnamic acid part as the enoyl-acyl backbone, following Lipinski's rules, attachment of drugs and drug-like molecules to the acid functionality (Figure 1). Variations were accomplished with the choice of modified cinnamic acids, drugs and drug-like molecules.

**Scheme 1:**
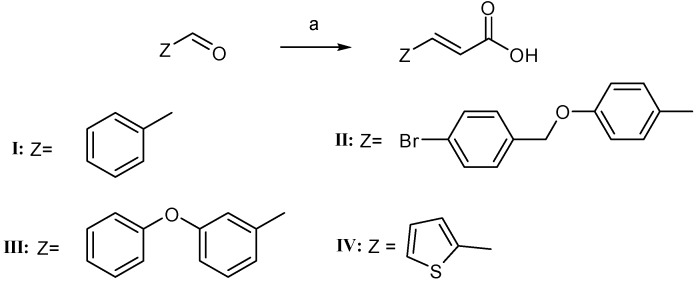
Synthesis of cinnamic acids.

The synthesis of cinnamic acids **I**–**IV** was established by a Knoevenagel-Doebner condensation of the suitable aldehyde with malonic acid in the presence of pyridine and piperidine, as shown in Scheme 1 and reported earlier [10,11,12,13,14]. The physicochemical and spectroscopic data of the obtained acids are identical to those given in the literature [10,11,12,13,14,16].

*Reagents and Conditions*: (a) pyridine; CH_2_(COOH)_2_; piperidine; reflux; 120–140 °C; 4 h.

**Scheme 2:**
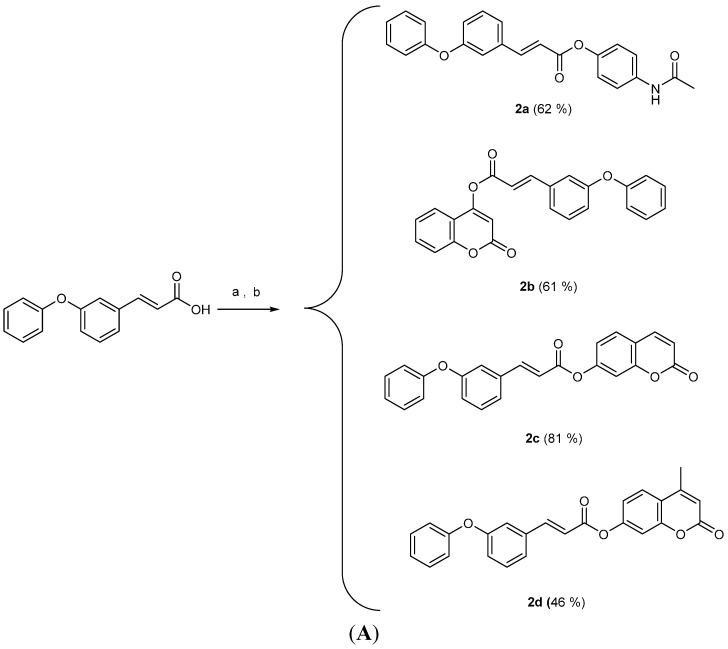

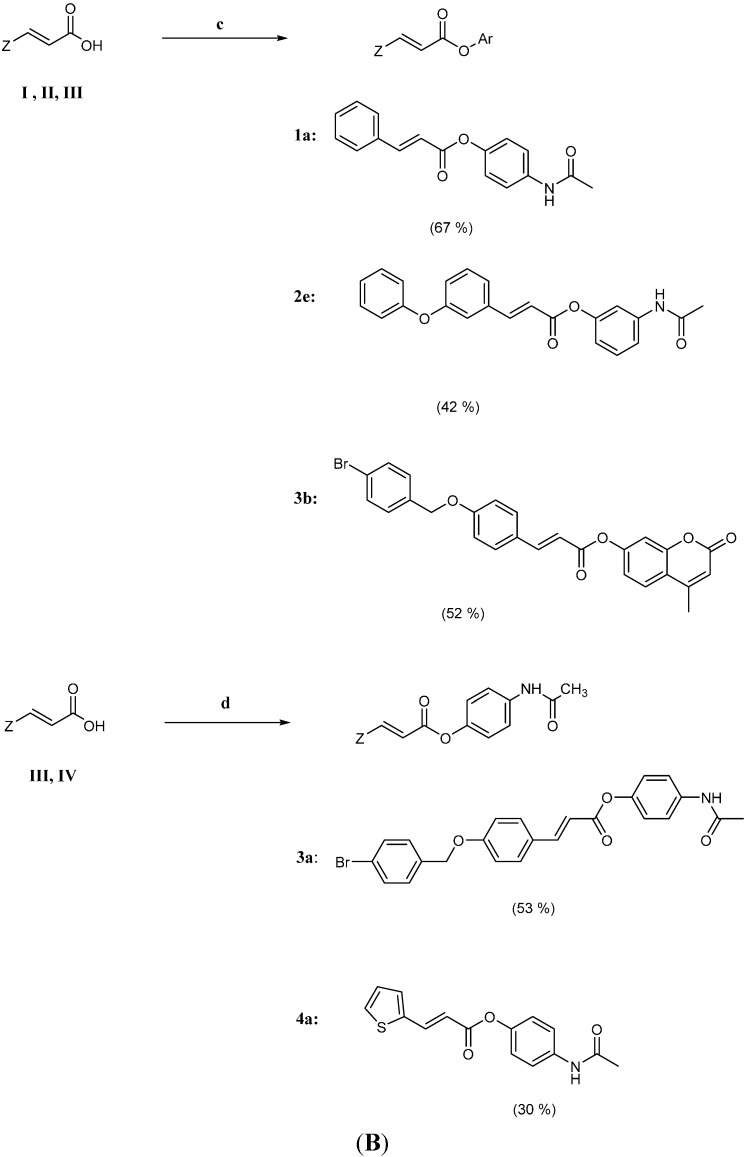
(**A**) Synthesis of cinnamic esters. (**B**) Synthesis of cinnamic esters.

The synthesis of hybrid cinnamic esters **2a**–**d** proceeded through the reaction of the corresponding cinnamoyl chloride and of a suitable substituted hydroxy-compound e.g., paracetamol, 4-hydroxy-coumarin, 7-hydroxycoumarin or 4-methyl-7-hydroxycoumarin) in an aqueous solution of sodium hydroxide (Scheme 2A). The synthesis of esters **3a** and **4a** followed a one pot procedure in anhydrous DMF, with triethylamine and *O*-(benzotriazol-1-yl)-*N,N,N',N''*-tetramethyluronium hexafluorophosphate (BOP) (Scheme 2B). Hybrid cinnamic esters **1a**, **2e** and **3b** were also derived from one pot synthesis between the appropriate acid **I**, **II** or **III** and a hydroxy-substituted compound—paracetamol (4-acetamidophenol), 3-acetamidophenol or hymechromone (4-methyl-7-hydroxycoumarin)—in dichloromethane, BOP and triethylamine (Scheme 2B).

**Scheme 3:**
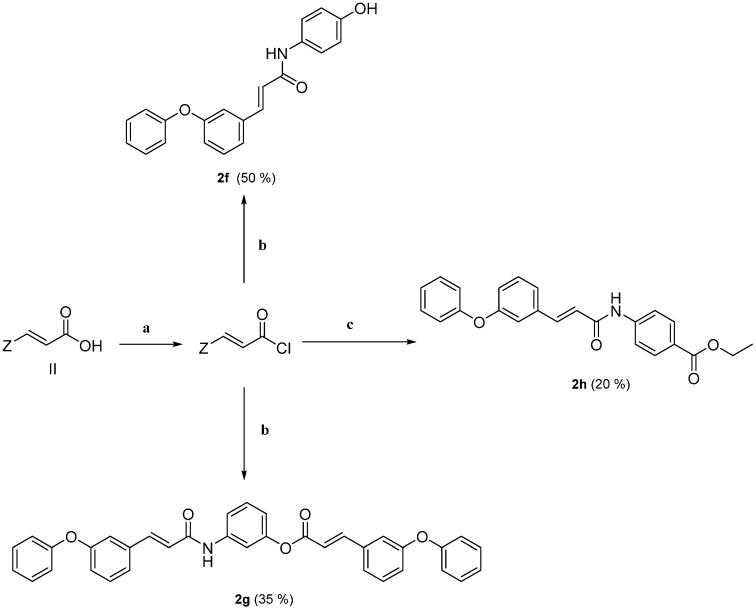
Synthesis of cinnamic amides.

The synthesis of hybrid cinnamic amide **2f** was accomplished by the reaction of *p*-aminophenol in an aqueous solution or suspension of sodium hydroxide with a solution of cinnamoyl chloride of acid **II** in dichloromethane. Similarly, the cinnamic amide **2h** was synthesized through the cinnamoyl chloride of acid **II** by the reaction of Benzocaine in the presence of pyridine (Scheme 3). The hybrid **2g** is a combination of an amide and an ester derived from the reaction with *m*-aminophenol.

The final products were obtained in good yields (30%–80%), with the only exception of compound **2h**, which was obtained in lower yields (20%). The pure final products were recrystallized from appropriate solvents or subjected to preparative thin layer chromatography (PLC). IR, ^1^H-NMR, ^13^C-NMR and elemental analysis were used for the confirmation and purity of the synthesized compounds structures. All the esters and amides present the characteristic absorption in the IR (nujol or KBr disk) 1720 (C=O), 1625 (C=C), cm^−1^) and correspond to the *E*-isomers (*J* > 9 Hz). The ^1^H-NMR and ^13^C-NMR data confirm the proposed structures. The LC-MS(ΕSΙ) examination points to [M+1]^+^ as well as to [M+1+Νa]^+^, [M+1+Κ]^+^, [M+1+Νa+MeOH]^+^. The synthesis of compound **1a** has been previously reported [17].

*Reagents and Conditions*: (a) SOCl_2_; reflux at 80 °C; (b) aq. NaOH/ H_2_O; CH_2_Cl_2_; 1.5 h; stirring conditions r.t. (c) CH_2_Cl_2_/Et_3_N; BOP- reagent; stirring at r.t. for 48 h; (d) DMF/Et_3_N; BOP-reagent; stirring at 0 °C for 15 min and stirring at r.t. for 2–4 h.

*Reagents and Conditions*: (a) SOCl_2_; reflux at 80 °C; (b) aq. NaOH/H_2_O; CH_2_Cl_2_; 1.5 h; stirring, r.t.; (c) pyridine; CH_2_Cl_2_; reflux 4 h.

### 2.2. Physicochemical Studies

Since lipophilicity is a significant physicochemical property determining distribution, bioavailability, metabolic activity and elimination, we tried to determine experimentally the lipophilicity of the new substances by a RPTLC method as R_M_ values (Table 1) and to compare them with the corresponding theoretically calculated clog *p* values in *n*-octanol-buffer [18]. This is considered to be a reliable, fast and convenient method for expressing lipophilicity [19]. Lipophilicity (Table 2), was theoretically calculated as Clog *p* values in *n*-octanol-buffer by the CLOGP Programme of Biobyte Corp. [20].

From our results (Table 1 and Table 2) and Equation (1), it can be concluded that R_M_ values could be used as a successful relative measure of the overall lipophilic/hydrophilic balance of these molecules:

R_M_ = 0.279 (0.111) clog *P* − 1.528 (0.677)

n = 9, r = 0.914, r^2^ = 0.836, q^2^ = 0.663, s = 0.271, F_1,7_ = 35.75, α = 0.01 (1)

Molar refractivity of the Ar substituents (MR-Ar) was theoretically calculated by the CLOGP Programme of Biobyte Corp. (Table 2) [20].These physicochemical properties were used in order to better describe the activity of the compounds in quantitative structure activity relationships (QSARs) [21].

**Table 1:** Lipophilicity values (R_M_) of cinnamic hybrids; % inhibition of lipid peroxidation (AAPH %); *in vitro* soybean lipoxygenase inhibition (IC_50_ μM or % LOX Inh.); *in vitro* trypsin induced proteolysis inhibition (IC_50_ or % Trypsin Inh.); *in vivo* analgesic-antinociceptive activity (% ANA); *in vivo* carrageenin-induced rat paw oedema inhibition (ICPE %).

Compounds	R_M_ (±S.D.) ^a^	AAPH % ^b,c^	IC_50_ μM ^c^ LOX Inh.	IC_50_ μM/% ^c^ Trypsin Inh.	% ANA (0.01 mmol/0.1 kg) ^d^	% ICPE (0.01 mmol/1 kg) ^e^
**I [18]**	−0.485 ± 0.044	78	56 μM	55 μM	60.2 *	nt
**II [15]**	−0.41 ± 0.026	84	66 μM	na	67 *	41.6 *
**III [15]**	−0.17 ± 0.016	84	89 μM	na	15.3 *	44.4 *
**IV [13,14,15,16,17]**	−0.56 ± 0.019	60	98 μM	na	nt	42.7 *
**1a**	−0.725 ± 0.009	95	10 μM	34% ^b^	77.3 **	nt
**2a**	0.276 ± 0.006	90	100 μM	10 μM	91 **	36.5 **
**2b**	−0.661± 0.008	100	100 μM	100 μM	nt	nt
**2c**	0.444 ±0.008	93	20% ^b^	10 μM	70.4 **	nt
**2d**	0.558 ± 0.040	97	74 μM	10 μM	66 **	nt
**2e**	0.407 ± 0.015	60	0.34 μM	na	98.1 **	nt
**2f**	−0.116 ± 0.003	100	na	26 μM	nt	nt
**2g**	1.092 ± 0.044	60	72 μM	43.6 μM	nt	nt
**2h**	0.399 ± 0.045	64	100 μM	50 μM	nt	nt
**3a**	−0.24 ± 0.009	96	55 μM	100 μM	52 **	nt
**3b**	−0.602 ± 0.01	90	50 μM	5 μM	nt	nt
**4a**	−0.826 ± 0.05	95	16% ^b^	na	nt	nt
**NDGA**	nt	nt	51.5 μM	nt	nt	nt
**Trolox**	nt	63	nt	nt	nt	nt
**Coumarin**	nt	nt	nt	13%	nt	nt
**4-HC**	nt	100	nt	na	nt	62 *
**7-HC**	nt	92	nt	100 μM	nt	nt
**Hymechromone**	nt	93	nt	60%	nt	nt
**Salicylic acid**	nt	nt	nt	53.6%	nt	nt
**Paracetamol**	nt	91	82 μM	na	66 *	21 *
**p-AP**	nt	99	nt	87%	nt	nt
**m-AP**	nt	95	nt	90%	nt	nt
**Benzocaine**	nt	25	nt	28%	nt	nt
**Indomethacin**	nt	nt	nt	nt	nt	47 *

na: no activity under the reported experimental conditions; nt: not tested; ^a^ R_M_ values are the average of at least five measurements; ^b^ % inhibition at 100 μM; ^c^ Values are means (±SD < 10%) of three or four different determinations. Means within each column differ significantly (*p* < 0.05); ^d^ Each value represents the mean obtained from 5 to 7 animals (±SD < 10%); * *p* < 0.1 ** *p* < 0.01 (Student’s *t*-test) in comparison to controls; ^e^ Each value represents the mean obtained from 6 to 15 animals in two independent experiments. In all cases, significant difference from control: * *p* < 0.1 ** *p* < 0.01 (Student’s *t*-test).

**Table 2:** Physicochemical data of cinnamic acid derivatives. 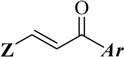

Compounds	Clog *P* ^a^	MR-*Ar*
**I**	2.24	-
**II**	3.28	-
**III**	4.34	-
**IV**	5.38	-
**1a**	3.28	3.92
**2a**	5.38	3.92
**2b**	6.02	4.06
**2c**	5.89	4.06
**2d**	6.39	4.52
**2e**	5.54	4.07
**2f**	5.10	2.74
**2g**	10.14	9.99
**2h**	6.73	4.54
**3a**	5.83	3.92
**3b**	6.84	4.52
**4a**	2.93	3.92

^a^ Theoretically calculated clog *p* values [20].

### 2.3. Biological Evaluation Antioxidant, Analgesic and Anti-Inflammatory Activity

Taking into account the multifactorial character of oxidative stress and inflammation, and the biological role of cinnamic acids **I**–**IV** [10,11,12,13,14,16] as well as of paracetamol, 4-/7-hydroxycoumarins, 4-methyl-7-OH-coumarin, *p*-aminophenol, *m*-aminophenol and benzocaine, we decided to evaluate in the present investigation the hybrids **1a**, **2a**–**h**, **3a**–**b** and **4a** with regard to their antioxidant ability as well as to their ability to inhibit soybean LOX and trypsin.

Many non-steroidal anti-inflammatory drugs have been reported to act either as inhibitors of free radical production or as radical scavengers [22]. Antioxidants acting as lipid peroxidation inhibitors could offer for health’s maintenance and to the compensation of risk factors [23].

Since the antioxidant ability of a compound is influenced by the experimental conditions, we have used two different assays to measure *in vitro* antioxidant activity: (a) the reduction of the stable free radical DPPH, (b) the reduction of the water-soluble azo compound 2,2-azo-bis(2-amidinopropane)-dihydrochloride (AAPH).

All the tested hybrids along with nordihydroguaeretic acid, as a reference compound, were observed visually for scavenging of DPPH and the presence of white spots on a purple background support their antioxidant ability.

The use of AAPH is recommended as more appropriate for measuring radical-scavenging activity *in vitro*, because the activity of the peroxyl radicals produced by the action of AAPH shows a greater similarity to cellular activities such as lipid peroxidation [24]. All the hybrids with the exception of **2e**, **2g** and **2h**, present high anti-lipid peoxidation activity (90%–100%), significantly higher than the corresponding acids **I**–**IV**. The presence of the cinnamoyl group is correlated with the activity which is influenced by the structural modifications of substituent Ar.

Regression analyses of the values of anti-lipid peroxidation AAPH% revealed that the molar refractivity of substituent Ar, is the main physicochemical parameter influencing their inhibitory activity. The negative sign points to a steric hindrance:

Log % AAPH = −0.032 (0.006) MR-Ar + 2.103 (0.031)
*n* = 10, r = 0.972, r^2^ = 0.946, s = 0.016, F_1,8_ = 140, α = 0.01 (2)

The cinnamic hybrids are expected to offer inhibition of LOX since their precursors cinnamic acids are potent LOX inhibitors. Lipoxygenases (LOXs) catalyze the first two steps in the metabolism of arachidonic acid to leukotrienes, important inflammatory mediators [25]. Study of LOX IC_50_ inhibition values demonstrates that compound **2e** is by far the most active inhibitor, followed by compounds **1a** and **3b** (Table 1) as well hybrids **3a** > **2g** > **2d**. It seems that these hybrids are much more potent compared to their precursors. Compounds **2c**, **2f** and **4a** exhibit low or no activity. Regression analyses of the values of IC_50_ revealed that the lipophilicity of the molecules as R_M_ values governs the biological response. The negative sign denotes that hydrophilicity (low lipophilicity) leads to higher inhibition:

Log 1/IC_50_ (LOX) = −2.822 (1.477) R_M_ + 2.845 (0.653)
*n* = 7, r = 0.910, r^2^ = 0.82, s = 0.601, F_1,5_ = 3.45, α = 0.1 (3)

Lipophilicity is referred to as an important physicochemical property for LOX inhibition [15]. The most potent hybrids **2e** and **1a** seems to follow this concept (clog*p* values for **2e**: 5.54 and for **1a**: 3.28). However, herein the role of lipophilicity is not well defined, since highly lipophilic compounds **2g**, **2h** and **2d** seemed less active than compounds **2e** and **1a** (Table 1 and Table 2). This goes in parallel to the above mentioned QSAR model (2).

Compounds **2a**, **2b** and **2h** are equipotent (IC_50_ = 100 µM). They all are hybrids of the same acid **II**. Considering the structural characteristics it seems that the esters are more potent inhibitors than the corresponding amides (**2e**, **3b**, **3a**, **1a**). Most of the LOX inhibitors are antioxidants or free radical scavengers [26]. Herein it seems that LOX inhibition is correlated with the anti-lipid peroxidation activity, with some exceptions (**4a**, **2g**, **2c**, **2e**).

We evaluated the ability of our hybrids as well as of their precursors to inhibit trypsin, a well known protease implicated in inflammation and tissues damage. Compound **3b** is a highly potent inhibitor (IC_50_ = 5 µM), a hybrid of bromobenzyloxy cinnamic acid **III** with paracetamol. It is followed by **2a**, **2c** and **2d** which all present equipotent activity (10 µM) and they are hybrids of the same acid **II**. In general all the hybrids exhibit anti-proteolytic activity higher than the corresponding precursors acids (only acid **I** presents inhibitory activity), with the exception of **1a** (34%) and **4a** which show low or no activity. Again the esters are more potent. The presence of a coumarin group is correlated with higher activity. It is interesting to note that **2g** which has an ester and an amide bond presents an interesting combination of anti-LOX and anti-proteolytic activity.

Regression analyses of IC_50_ values revealed that the molar refractivity of substituent Ar and the overall lipophilicity as clog*p* values of the molecule are the most significant physicochemical properties influencing the anti-proteolytic activity. The negative sign of MR points to steric hindrance:

Log 1/(IC_50_)= −0.131 (0.08) MR-Ar + 0.148 (0.08) ClogP + 1.486 (0.288)
*n* = 10, r = 0.858, r^2^ = 0.736, s = 0.115, F_2,7_ = 1.94, α = 0.1 (4)

In compounds **2a** and **2b** the ester group has been hydrolyzed under the influence of an esterase. This could be attributed as a preliminary *in vitro* metabolic assay. Spectroscopic data and chromatographic analysis confirmed the cleavage of hybrid molecules under the given experimental conditions. The analytical data confirmed the stability of hybrid **2a** under acidic and basic conditions.

Hybrid **2a** with the most significant biological profile was tested for its anti-inflammatory activity using the carrageenin rat paw edema. The result is low (36.5%) compared to indomethacin (47%) but higher than paracetamol (21%) and close to acid **II** (44%).

**Figure 2:**
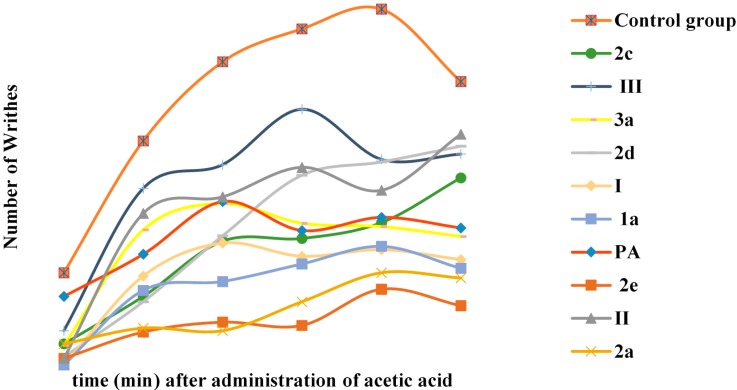
Antinociceptive activity of hybrids **1a**, **2a**–**e**, **3a**, of the corresponding cinnamic acids **I**, **II**, **III** and of p-acetamidophenol (PA) using the writhing test.

Since most of the non-steroidal anti-inflammatory agents possess analgesic properties (non-opioid), the antinociceptive activity of selected hybrids was examined [27] using the writhing test. [28] (Table 1, Figure 2).Taking under consideration the *in vitro* results, hybrids with significant multifunctional activities were chosen to be tested *in vivo* for their analgesic activity. Another criterion for the selection was the combination with paracetamol, which is generally considered as an NSAID which demonstrates potent anti-inflammatory, antipyretic and analgesic activity.

We did not evaluate acid **IV** and hybrid **4a** since the later was almost inactive according to the *in vitro* tests. From the tested compounds, it was found that hybrid **2e** highly and significantly inhibited the acetic acid-induced writhing (98.1%, Table 1), whereas compounds **2a**, **1a**, **2c** and **2d** followed. The tested acids **I** and **III** present lower activity than their derived hybrids. The hybrids of series **2** are more potent than acid **II** with the exception of **2d** which is almost equipotent to **II**. The analgesic activity was observed to be constantly high during the observation period of 30 min.

Perusal of Table 1 shows that anti-inflammatory activity and antinociception do not proceed in parallel. This however is not unexpected, since the case of aspirin and paracetamol is similar [29,30]. Recent studies [31,32] suggest the potential utility of NSAIDs in the development of new therapeutic approaches to treat peripheral nerve injury. It seems likely that the eicosanoid, opioidergic, serotonergic and cannaboid system might be responsible for the neuroprotection [33] and recovery of motor and sensory functions, when acetaminophen is administered immediately after the compression of sciatic nerve in rat. Sciatic nerve crush in rats represents a predictive model to study molecules with a therapeutic potential in human neuropathic pain syndromes. In addition, it has been reported that in this model peripheral neuropathy is associated to a reduction of central 5-HT release. It has been also found that paracetamol reduces neuropathic pain-like behaviour in rats by potentiating 5-hydroxy-tryptamine (serotonin e 5-HT) neurotransmission and reinforced the role of central serotonergic systems in nociceptive processing [34].

**Figure 3:**
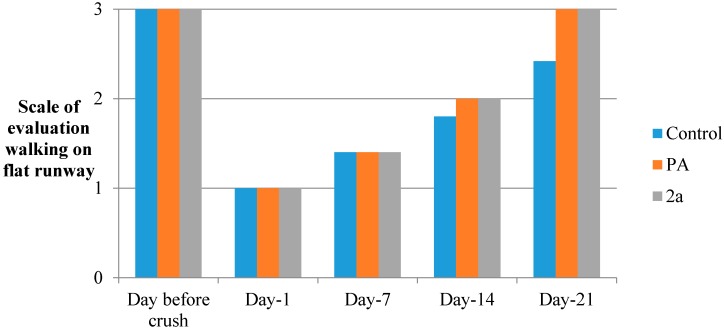
Flat runway with S.D. < 10%.

**Figure 4:**
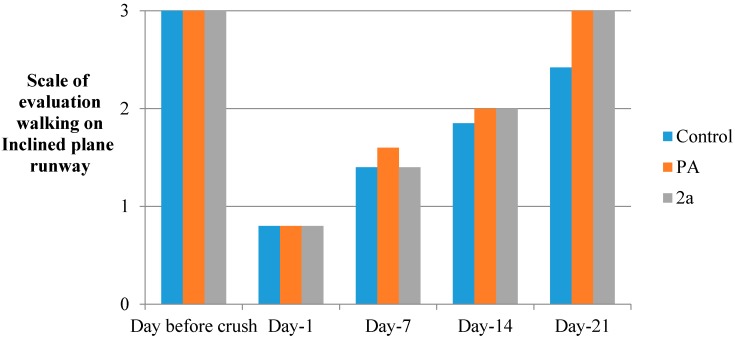
Inclined plane runway (S.D. < 10%).

**Figure 5:**
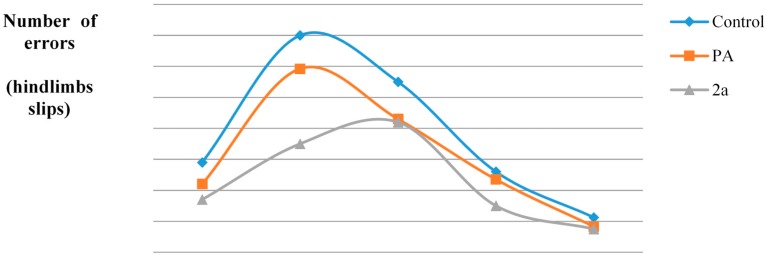
Grid runway (S.D. < 10%).

**Figure 6:**
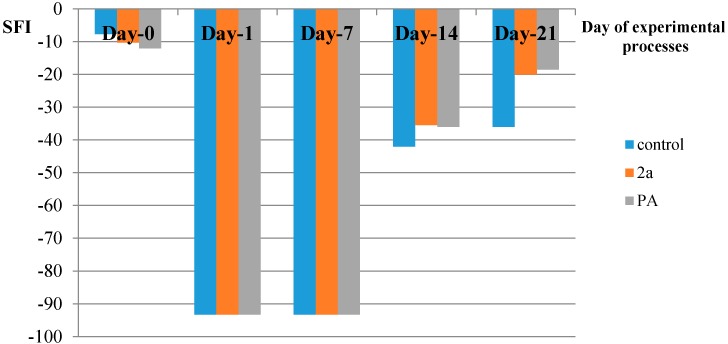
Sciatic Functional Index (SFI) (S.D. < 10%).

**Figure 7:**
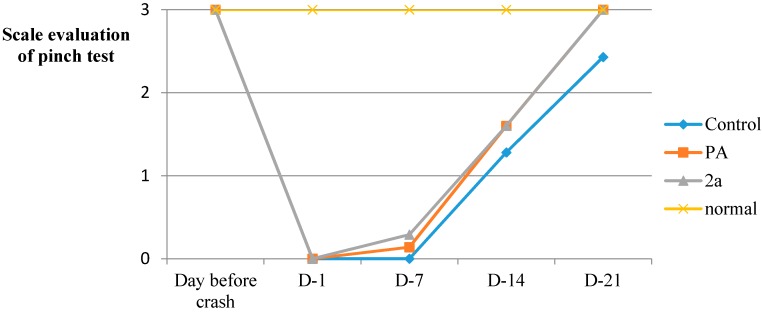
Pinch test (S.D. < 10%).

In the current study, the hybrid of paracetamol **2a** was shown to be effective in recovering motor and sensory functions [35] in adult rats after sciatic nerve crush, when administered intraperitoneally immediately after the compression of the nerve (Figure 3, Figure 4, Figure 5, Figure 6 and Figure 7 and Table 3). The locomotor function recovery was examined through the flat runway test, the inclined plane runway test, the grid walking test and the Sciatic Functional Index (SFI) calculation. The two runway tests conducted were evaluated with the following modified score technique, showed in Table 3 [36,37].

**Table 3:** Evaluation of behavior of rats on different runways.

Score	Movement in Hindlimb
0	No movement
1	Mild movement
2	Walking with mild deficit
3	Nearly normal

The results from the flat runway test showed a very significant improvement of the treated with **2a** crushed animals. This improvement is observed from day 1 to day 21. The behavior of the rats under treatment seems to be nearly normal (3) compared to the healthy and untreated controls (Figure 3).

Judging the results from the inclined plane runway test, the treated rats seems to be able to balance their body position compared to the controls (Figure 4). The results from the grid runway test point to the fact that the treated animals succeeded to walk on the grid without errors (hindlimb slips) from day 1 to day 22 in comparison to the controls (Figure 4).

SFI is the current standard for measuring functional recovery following rat sciatic nerve crush. Its calculation involves the measurement of wide-ranging relationships between feet and toes of the hindlimb of recovering animals. SFI values close to −100 indicate severe damage, while SFI values close to 0 indicate normal function. The treated rats improved their walk and the relationship between feet and toes of the crushed hindlimbs starting from day 14 to day 21. The SFI values are given in Table 4 and Figure 6.

**Table 4:** Values of Sciatic Functional Index.

SFI	Day before Crash	Day-1	Day-7	Day-14	Day-21
Control	−7.67	−93.3	−93.3	−42	−36
PA	−12	−93.3	−93.3	−36	−18.5
2a	−10.26	−93.3	−93.3	−35.45	−20.06

Recovery of sensory function was analyzed using the pinch test. At midthigh, the sciatic nerve is composed of about 27,000 axons. 71% are myelinated and unmyelinated sensory axons while only 6% are myelinated motor axons and 23% are sympathetic unmyelinated axons. [38] Therefore, control of the sensation of pain provides information about the degree of sciatic nerve regeneration. The improvement of the treated crushed animals is indicated within the results of Figure 7. The recovery of sensation is absolutely comparable to the healthy controls on the 21st day started from the 14th day.

Our results seem to be in parallel to the findings of paracetamol [35]. This preliminary study shows that hybrid **2a** could be a promising agent for treating peripheral nerve injuries. Further investigations are in progress to show the involvement of other systems/factors in the regulation of motor and sensory functions induced by **2a**.

### 2.4. Computational Studies

#### 2.4.1. Computational Methods, Docking Simulations

All the molecules were constructed with the ChemDraw program [39] and converted into 3D-Structures with the OpenBabel program [40], by using MMFF94 force field. Protein setup was performed using the UCSF Chimera software [41,42]. The AnteChamber PYthon Parser interfacE (ACPYPE) tool [43] was employed to generate the topologies of the ligands. ACPYPE tool is written in python to use Antechamber [44,45] to generate topologies for chemical compounds was used for the parametrization of the ligands. Energy minimizations where carried out with the molecular simulation toolkit GROMACS [46] using the AMBER99SB-ILDN force field [47].

Docking calculations were performed with the software Autodock Vina [48]. The PyRx program [49] was employed to generate the docking input files and to analyze the docking results. The proteins were considered rigid. Performing a blind docking the protein 1RRH soybean LOX, acid II, paracetamol and hybrid **2a** were properly aligned in the binding site of LOX. For all the ligands, the single bonds were considered as active torsional bonds. Docking was carried out with an exhaustiveness value of 64 and a maximum output of 100 binding modes. The results of ligands from protein 1RRH presented identical conformation in the binding site of 1RRH as they were crystallized in 1IK3. The final output of the docking procedure is a set of solutions ranked according to the corresponding scoring function values, each defined by the 3D coordinates of its atoms and expressed as a PDB file.

#### 2.4.2. Molecular Docking Studies on Soybean Lipoxygenase

The lack of structural data for human LOX, led us to model human LOX using soybean enzyme because of its availability and its well characterized structure [50]. The differences or similarities in activity toward soybean LOX, as well as the possible mechanism of action could be explained by docking calculations. Docking studies were performed for hybrid **2a** due to its overall significant biological profile in correlation with its *in vitro/in vivo* results, as well as for acid II and paracetamol, for the sake of comparison.

For the docking studies of LOX we have used the 1RRH (soybean lipoxygenase) available from the Protein Data Bank (PDB) with a resolution of 2 Å [51]. We used the 1RRH from PDB with Fe^+3^ running blind docking. Two soybean LOX models were derived from 1RRH. One with a ligand taken from 1IK3 (ligand ID: 9OH) for the docking studies into the catalytic cavity and another one without any ligand in the catalytic cavity for the blind docking simulation. The metal center in both models has been considered as a cation, with charge q = +3 and no bond restraint was applied between the iron and the ligands. Lennard-Jones parameters for Fe (III) force field can be resumed as: σ_vdw_ = 2.138157e^−1^ nm, ε_vdw_ = 2.092e^−1^ kJ/mol. The validation of our docking study was made with the ligands crystallized in the protein of complex 1IK3 where our docking results were in good agreement with the structure of the molecules in the active site of the protein.

**Figure 8:**
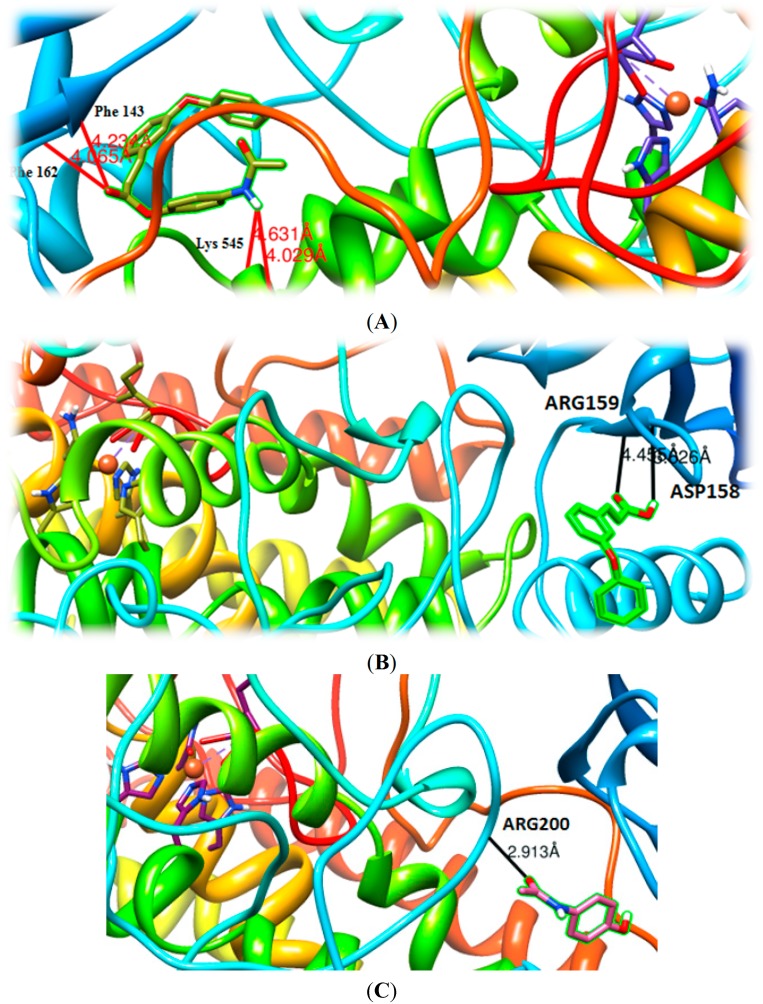
(**A**) Docked poses of hybrid 2a (in the LOX binding site. Ball and stick models are used to render the side-chains of relevant binding site residues. The iron ion is depicted as an orange sphere; (**B**) Docked poses of acid II in the LOX binding site. Ball and stick models are used to render the side-chains of relevant binding site residues. The iron ion is depicted as an orange sphere; (**C**) Docked poses of paracetamol in the LOX binding site. Ball and stick models are used to render the side-chains of relevant binding site residues. The iron ion is depicted as an orange sphere.

Molecular docking studies were performed on all the new synthesized hybrid compounds as well as on paracetamol and on acids. The docking orientations of acid **II**, paracetamol and hybrid **2a** are given in Figure 8A–C. For the presentation herein, we selected hybrid **2a** (Figure 8A) due to its overall significant biological profile (in correlation with its *in vitro/in vivo* results) and the best modeling results, as well as acid **II** and paracetamol, for the sake of comparison. Hybrid **2a** and and acid **II** are bound to the same area of the protein. However, hybrid **2a** presents different binding mode than acid **II** and higher binding energy to the protein (−9.8 kcal/mol) compared to the acid **II** (−6.9 kcal/mol).

A number of H-bonds were developed between the esteric carbonyl oxygen of **2a** with Phe143 and Phe162 and the amide oxygen with Lys545. The enzyme-**2a** complex it seems to be further stabilized by hydrophobic interactions with the aromatic rings of compound **2a** which are highly stronger than the hydrophobic interactions of acid **II**. Hybrid **2a** is well fitted in another area of the protein and not to the central cavity of LOX. Probable reason for this could be its bulk, since the angular structure is not flexible enough for the enzyme’s cavity. From the above, the possibility of an allosteric effect might be considered.

According to the docking data, it seems that the dinding site of hybrid **2a** is far from the active site of the enzyme. Furthermore, blockage of the cavity entrance might disturb the access to the catalytic site. The acid **II** is bound in the same area with the hybrid **2a** and showed hydrogen bonds between the carboxylic group and Asp158 and Arg159 (Figure 8B). Although the hydrogen bonds of these two compounds seem to be quite similar in strength, the hydrophobic bonds as well as the angular structure of compound **2a** seem to play the most crucial role for the higher inhibitory activity.

As it is shown in Figure 8C, paracetamol is bound to a different area of LOX with a significantly lower binding energy (−5.9 kcal/mol). Strong hydrogen bonds were developed between hydroxyl group and Arg200. The hydrogen bonds do not seem to be strong enough to support higher inhibitory activity and this is in accordance to our previous studies, which indicated the crucial role of hydrophobic interaction of ligand to lipoxygenase binding site [52].

## 3. Experimental Section

### 3.1. Materials and Instruments

All chemicals, solvents, chemical and biochemical reagents were of analytical grade and purchased from commercial sources (Merck, Merck KGaA, Darmstadt, Germany, Fluka Sigma-Aldrich Laborchemikalien GmbH, Hannover, Germany, Alfa Aesar, Karlsruhe, Germany and Sigma, St. Louis, MO, USA). Soybean lipoxygenase, pancreatic bovine trypsin, sodium linoleate, free radical DPPH and 2,2-azinobis-2-methyl-propanimidamine HCl (AAPH) were obtained from Sigma Chemical, Co. (St. Louis, MO, USA), carrageenin, type K, was commercially available. All starting materials were obtained from commercial sources and used without further purification.

Melting points (uncorrected) were determined on a MEL-Temp II (Lab Devices, Holliston, MA, USA). For the *in vitro* tests, UV-Vis spectra were obtained on a Perkin-Elmer 554 double beam spectrophotometer (Perkin-Elmer Corporation Ltd., Lane Beaconsfield, Bucks, England), were used. Infrared spectra (film as Nujol mulls or KBr pellets) were recorded with Perkin-Elmer 597 spectrophotometer (Perkin-Elmer Corporation Ltd., Lane Beaconsfield, Bucks, England). The ^1^H-NMR spectra were recorded at 300 MHz on a Bruker AM-300 spectrometer (Bruker Analytische Messtechnik GmbH, Rheinstetten, Germany) in CDCl_3_ or DMSO using tetramethylsilane as an internal standard unless otherwise stated. ^13^C-NMR spectra were obtained at 75.5 MHz on a Bruker AM-300 spectrometer in CDCl_3_ or DMSO solutions with tetramethylsilane as internal reference unless otherwise stated. Chemical shifts are expressed in δ (ppm) and coupling constants *J* in Hz. Mass spectra were determined on a LC-MS 2010 EV Shimadzu (Shimadzu Scientific Instruments, Inc., Columbia, MA, USA), using MeOH as solvent. Elemental analyses for C and H gave values acceptably close to the theoretical values (±0.4%) in a Perkin-Elmer 240B CHN analyzer (Perkin-Elmer Corporation Ltd., Lane Beaconsfield, Bucks, UK). Reactions were monitored by thin layer chromatography on 5554 F_254_ Silica gel/TLC cards, Merck and Fluka Chemie GmbH Buchs, Steinheim, Switzerland. For preparative thin layer chromatography (PLC) Silica gel 60 F_254_, plates 2 mm, Merck KGaA ICH078057 were used. For the experimental determination of the lipophilicity using reverse phase thin layer chromatography (RPTLC) TLC-Silica gel 60 F_254_ DC Kieselgel, Merck (20 × 20 cm) plates were used.

### 3.2. Chemistry General Procedure

#### 3.2.1. General Procedure for the Synthesis of Hybrid Cinnamic Esters **2a**–**d** (through the Formation of Cinnamoyl Chloride)]

The synthesis of cinnamic acids **I**–**IV** is accomplished by Knoevenangel-Doebner reaction between the appropriate aldehyde and malonic acid in the presence of piperidine and pyridine and it has been reported earlier [10,11,12,13,14,15,16]. A mixture of the corresponding substituted cinnamic acid **II** (0.83 mmol) and thionyl chloride (3.5 mmol) was stirred under reflux. Reaction was monitored by thin-layer chromatography (TLC), on aluminum cards precoated with 0.2 mm of silica gel and fluorescent indicator. When the reaction was finished (4 h approximately), the produced cinnamoyl chloride was used immediatelly for the next step without any further purification.

To a chilled solution containing cinnamoyl chloride (0.83 mmol) and dichloromethane (0.83 mL) was added dropwise over a period of 30 min with stirring, an aqueous solution of sodium hydroxide (1.7 mmol) and of a suitable substituted hydroxy compound (1.7 mmol, paracetamol or 4-hydroxy-coumarin or 7-hydroxycoumarin or 4-methyl-7-hydroxycoumarin) [53]. The mixture was stirred additionally for 1.5 h at room temperature. The product was obtained by filtration, washed with ice water and recrystallized from aqueous ethanol.

*(E)-4-Acetamidophenyl 3-(3-phenoxyphenyl)acrylate* (**2a**). Yield: 62%; R_f_ (ethyl acetate): 0.7; m.p.: 191–195 °C; IR (KBr, cm^−1^): 3355.8, 1718.2, 1695.9, 1671.2; LC-MS (*m/z*): (C_23_H_19_NO_4_) [M+Na]^+^ = 396, [M+K]^+^ = 412, [M+Na+MeOH]^+^ = 428; ^1^H-NMR (CDCl_3_, δ): 2.17 (s, 3H, CH_3_), 6.52–6.57 (d, 1H, *J* = 15 Hz), 7.02–7.05 (d, 2H, *J =* 9 Hz), 7.09–7.13 (m, 3H, aromatic), 7.14–7.20 (m, 2H, aromatic), 7.37–7.19 (m, 4H, aromatic), 7.5–7.53 (d, 2H, *J =* 9 Hz), 7.77–7.82 (d, 1H, *J =* 15 Hz); ^13^C-NMR (CDCl_3_, δ): 77, 107.4, 116.8, 117.8, 119.3, 120.9, 122, 123.2, 129.9, 130.3, 145.9, 156.7, 158.1, 189.9, 203.7; Analysis C, H, N. Expected %: (C_23_H_19_NO_4_) C: 73.98, H: 5.13, N: 3.75; Found%: C: 74.01, H: 5.09, N: 3.58.

*(E)-2-Oxo-2H-chromen-4-yl 3-(3-phenoxyphenyl)acrylate* (**2b**). Yield: 61%; R_f_ (ether/ethanol 1:1): 0.8; m.p.: 142–145 °C; IR (KBr, cm^−1^): 3071.2, 1746.8, 1704, 1690.8, 1614.6; LC-MS (*m/z*): (C_24_H_16_O_5_) [M+H]^+^ = 385, [M+Na]^+^ = 407, [M+K]^+^ = 423, [M+Na+MeOH]^+^ = 439; ^1^H-NMR (CDCl_3_, δ): 6.57 (s, 1H), 6.59–6.64 (d, 1H, *J =* 15 Hz), 7.04–7.06 (d, 2H, *J =* 6 Hz, aromatic), 7.11–7.19 (dd, 2H, *J =* 12 Hz, aromatic), 7.28–7.3 (m, 2H,aromatic), 7.30–7.44 (m, 5H, aromatic), 7.59 (t, 1H, *J =* 16 Hz), 7.68 (d, 1H, *J =* 8 Hz), 7.91 (d, 1H, *J =* 15 Hz); ^13^C-NMR (CDCl_3_, δ): 105.32, 115.74, 116.29, 117.14, 118.02, 119.28, 121.64, 122.82, 123.5, 123.9, 124.28, 130, 130.5, 132.73, 135.4, 148.53, 158.33, 161.47, 162.6, 174.43 Analysis C, H, N. Expected %: (C_24_H_16_O_5_) C: 74.99, H: 4.20; Found %: C: 74.87, H: 4.34.

*(E)-2-Oxo-2H-chromen-7-yl 3-(3-phenoxyphenyl)acrylate* (**2c**). Yield: 81%; R_f_ (methanol/acetone 3:1): 0.7; m.p.: 135–141 °C; IR (KBr, cm^−1^): 3081.2, 2924.2, 1725.1, 1594.5; LC-MS (*m/z*):(C_24_H_16_O_5_) [M+1]^+^ = 385, [M+Na]^+^ = 407, [M+K]^+^ = 423, [M+Na+MeOH]^+^ = 439; ^1^H-NMR (CDCl_3_, δ): 6.38–6.41 (d, 1H, *J =* 9 Hz), 6.53–6.59 (d, 1H, *J =* 15 Hz), 7.03–7.21 (m, 7H, aromatic), 7.3–7.42 (m, 4H, aromatic), 7.49–7.51 (d, 1H, *J =* 6 Hz), 7.69 (d, 1H, *J =* 9 Hz), 7.84 (d, 1H, *J =* 15 Hz); Analysis C, H, N. Expected %: (C_24_H_16_O_5_) C: 74.99, H: 4.20; Found %: C: 74.91, H: 4.35.

*(E)-4-Methyl-2-oxo-2H-chromen-7-yl 3-(3-phenoxyphenyl)acrylate* (**2d**). Yield: 46%; R_f_ (petroleum ether/ethyl acetate 1:3): 0.8; m.p.: 185–189 °C; IR (KBr, cm^−1^): 2927.4, 1734.8, 1607.9; LC-MS (*m/z*): (C_25_H_18_O_5_) [M+H]^+^ = 399, [M+Na]^+^ = 421, [M+K]^+^ = 437, [M+Na+MeOH]^+^ = 453; ^1^H-NMR (CDCl_3_, δ): 2.45 (s, 3H, CH_3_), 6.29 (s, 1H), 6.58 (d, 1H, *J =* 15 Hz), 7.04–7.11 (m, 3H, aromatic), 7.14–7.21 (m, 4H, aromatic), 7.31–7.34 (d, 1H, *J =* 9 Hz), 7.36–7.4 (m, 2H, aromatic), 7.63–7.66 (d, 1H, *J =* 9 Hz), 7.85 (d, 1H, *J =* 15 Hz); ^13^C-NMR (CDCl_3_, δ): 18.78, 110.48, 117.13, 117.71, 118.13, 119.26, 121.13, 123.27, 123.9, 129.95, 125.39, 130.38, 146.93, 158.04; Analysis C, H, N. Expected %: (C_25_H_18_O_5_) C: 75.37, H: 4.55; Found %: C: 75.41, H: 4.40.

#### 3.2.2. One Pot Synthesis of Hybrid Cinnamic Esters **3a** and **4a**

To an ice cold solution of cinnamic acid **III** or **IV** (2 mmol) and paracetamol (2.2 mmol) in anhydrous DMF, triethylamine (6.9 mmol) and *O*-(benzotriazol-1-yl)-*N,N,N',N''*-tetramethyluronium hexafluorophosphate (BOP, 2.3 mmol) were added. The resulted mixture was stirred for 15 min at 0 °C. Stirring was continued at room temperature further for 1 h to complete the reaction (monitored by TLC). The obtained crude product was dissolved in CHCl_3_ and washed sequentially with 5% aqueous citric acid solution, H_2_O, 5% aqueous NaHCO_3_ solution and H_2_O. Then, it was dried over Na_2_SO_4_ and finally evaporated to dryness to leave a solid residue.[54] The pure final product is obtained by recrystallization from ethyl acetate initially and then from 95% aqueous ethanol.

*(E)-4-Acetamidophenyl 3-(4-((4-bromobenzyl)oxy)phenyl)acrylate* (**3a**). Yield: 53%; R_f_ (petroleum ether/ethyl acetate 1:3): 0.7; m.p.: 212–215 °C; IR (KBr, cm^−1^): 2725.2, 1774.3, 1597.7; LC-MS (*m/z*): (C_24_H_20_BrNO_4_) [M]^+^ = 466 (468), [M−O]^+^ = 450 (452); ^1^H-NMR (CDCl_3_, δ): 1.45 (s, 3H, -CH_3_), 5.02 (s, 2H, -CH_2_-O-), 6.5–6.55 (d, 1H, *J =* 15 Hz), 6.94–6.97 (d, 2H, *J =* 9 Hz), 7.22–7.35 (d, 2H, *J =* 9 Hz), 7.33–7.42 (m, 2H, aromatic), 7.45–7.54 (m, 4H, aromatic), 7.91–8.02 (m, 2H, aromatic, 1H vinyl hydrogen); Analysis C, H, N. Expected %: (C_24_H_20_BrNO_4_) C: 61.81, H: 4.32, N: 3.00; Found %: C: 66.87, H: 4.11, N: 3.38.

*(E)-4-Acetamidophenyl 3-(thiophen-2-yl)acrylate* (**4a**). Yield: 30%; R_f_ (toluene/ethyl acetate 1:1): 0.7; m.p.: 162–164 °C; IR (CHCl_3_, cm^−1^): 3681.2, 3019.3, 1700.4, 1650, 1603.9; LC-MS (*m/z*): (C_15_H_13_ NO_3_ S) [M−O]^+^ = 271, [M−O+1]^+^ = 272, [M−O+Na]^+^ = 294, [M−O+K]^+^ = 310, [M−O+Na]^+^ = 326; ^1^H-NMR (CDCl_3_, δ): 1.81 (s, 3H, -CH_3_), 5.29 (s, 1H, N-H), 7.15–7.20 (d, 1H, *J =* 15 Hz), 7.46–7.49 (d, 2H, *J =* 9 Hz), 7.54–7.57 (d, 2H, *J =* 9 Hz), 7.79 (m, 1H, aromatic), 8.02–8.05 (d, 1H, *J =* 9 Hz), 8.16–8.21 (d, 1H, *J =* 15 Hz), 8.51–8.54 (d, 1H, *J =* 9 Hz); Analysis C, H, N. Expected %: (C_15_H_13_ NO_3_ S) C: 62.7, H: 4.56, N: 4.87; Found %: C: 62.57, H: 4.55, N: 4.66.

#### 3.2.3. One Pot Synthesis of Hybrid Cinnamic Esters **1a**, **2e** and **3b**

To a stirred solution of the appropriate acid **1** or **2** (1.4 mmol) of paracetamol (4-acetamidophenol) or hymechromone (4-methyl-7-hydroxycoumarin, 1.5 mmol) in dichloromethane (6 mL), BOP (2.4 mmol) and triethylamine (5.6 mmol) were added. After 48 h stirring at room temperature, dichloromethane was removed under reduced pressure and ethyl acetate (80 mL) was added to the reaction mixture. The resulted solution was washed with 10% citric acid (2 × 20 mL), 10% solution of NaHCO_3_ (3 × 20 mL), water (20 mL) and brine (2 × 20 mL), dried over anhydrous sodium sulfate, filtered and concentrated under reduced pressure [17]. The crude product was purified by column chromatography or crystallized from the appropriate solvent.

*(E) 4-Acetamidophenyl cinnamate* (**1a**). The physicochemical and spectroscopic data of the obtained product are identical to those given in the literature [17].

*3-Acetamidophenyl (E)-3-(3-phenoxyphenyl)acrylate* (**2e**)*.* The crude product was purified by column chromatography with mixture of petroleum ether/ethyl acetate (1:1). Yield: 42%; R_f_ (ethyl acetate/petroleum ether 1:1): 0.5; m.p.: 112–114 °C; IR (KBr, cm^−1^): 2465.3, 1720.9, 1608.1; LC-MS (*m/z*): (C_23_H_19_NO_4_) [M+H]^+^ = 374, [M+Na]^+^ = 396, [M+K]^+^ = 412, [M+Na+MeOH]^+^ = 428; ^1^H-NMR (CDCl_3_, δ): 2.14 (s, 3H, -CH_3_), 6.55–6.60 (d, 1H, vinyl hydrogen, *J =* 15 Hz), 6.92–6.95 (d, 1H, *J =* 9 Hz), 7.06–7.11 (m, 3H, aromatic), 7.15–7.20 (m, 1H, aromatic), 7.29–7.38 (m, 7H, aromatic), 7.43–7.40 (d, 1H, *J =* 9 Hz), 7.80–7.85 (d, 1H, *J =* 15 Hz); ^13^C-NMR (CDCl_3_, δ): 24.51, 113.27, 116.96, 117.07, 117.73, 117.83, 119.13, 119.23, 120.91, 123.19, 123.84, 130.30, 129.97, 129.93, 129.58, 135.83, 139.14, 146.13, 150.97, 156.53, 158.01, 165.35, 168.42; Analysis C, H, N. Expected %: (C_23_H_19_NO_4_) C: 73.98; H, 5.13; Found %: C: 73.78, H: 4.89.

*(E)-4-Methyl-2-oxo-2H-chromen-7-yl-3-(4-((4-bromobenzyl)oxy)phenyl)acrylate* (**3b**). Yield: 52%; R_f_ (ethyl acetate/petroleum ether 3:1): 0.5; m.p.: 192–194 °C; IR (KBr, cm^−1^): 2370.5, 1724.8, 1601.3; LC-MS (*m/z*): (C_26_H_19_ BrO_5_) [M]^+^ = 491 (493), [M+Na]^+^ = 511 (513), [M+K]^+^ = 529 (531), [M+Na+MeOH]^+^ = 545 (547); ^1^H-NMR (CDCl_3_, δ): 2.43 (s, 3H, -CH_3_), 5.07 (s, 2H, -CH_2_-O-), 6.26 (s, 1H, vinyl hydrogen), 6.48 (d, 1H, *J =* 18 Hz), 6.97–7.00 (d, 1H, *J =* 9 Hz), 7.14–7.20 (m, 3H, aromatic), 7.28–7.31 (m, 2H, aromatic), 7.51–7.56 (m, 4H, aromatic), 7.62 (d, 1H, *J =* 9 Hz), 7.84 (d, 1H, *J =* 18 Hz); Analysis C, H, N. Expected %: (C_26_H_19_BrO_5_) C: 63.56, H: 3.9; Found %: C: 63.49, H: 3.90.

#### 3.2.4. General Procedure for the Synthesis of Hybrid Cinnamic Amides **2f**, **2g**

The synthesis of hybrid cinnamic amides **2f** or **2g** followed a two-step reaction [53]. Firstly the cinnamic acid chloride was prepared in the presence of thionyl chloride. To the stirred solution of cinnamoyl chloride (1 mmol, without further purification) in dichloromethane (1 mL) an aqueous solution or suspension of sodium hydroxide (2 mmol) and of *p*-aminophenol or *m*-aminophenol (2 mmol) was added slowly (15 min). The mixture was stirred additionally for 1.5 h at room temperature. The product **2f** was filtered, washed with ice water and recrystallized from ethyl acetate. The hybrid molecule **2g** was recrystallized from 95% ethanol, filtered and washed with diethylether. The hybrid product **2g** was further purified chromatographically (PLC) with a mixture of toluene-ethyl acetate (3:1).

*(E)-N-(4-Hydroxyphenyl)-3-(3-phenoxyphenyl)acrylamide* (**2f**). Yield: 50%; R_f_ (ethyl acetate/petroleum ether 1:1): 0.7; m.p.: 212–215 °C; IR (KBr, cm^−1^): 3446.2, 3312.4, 1659.3, 1628.1; LC-MS (*m/z*): (C_21_H_17_NO_3_) [M+H]^+^ = 332, [M−H]^+^ = 330 [M+Na]^+^ = 354, [M+K]^+^ = 370, [M+Na+MeOH]^+^ = 386; ^1^H-NMR (CDCl_3_, δ): 6.28 (s, 1H, N-H), 6.37 (d, 1H, *J =* 15 Hz), 6.72–6.75(d, 2H, *J =* 9 Hz), 6.94–6.97 (d, 2H, *J =* 9 Hz), 7.06–7.09 (m, 3H, aromatic), 7.16–7.18 (m, 2H, aromatic), 7.26–7.31 (m, 4H, aromatic), 7.6 (d, 1H, *J =* 15 Hz); ^13^C-NMR (DMSO-d_6_, CDCl_3_): 114.41, 116.2, 117.9, 118.77, 120.43, 122.17, 122.58, 128.94, 129.28, 130, 136.1, 138.22, 152.74, 155.78, 156.49, 162.64; Analysis C, H, N. Expected %: (C_21_H_17_NO_3_) C: 76.12, H: 5.17, N: 4.23; Found %: C: 76.09, H: 5.27, N: 3.89.

*(E)-3-(E)-3-(3-phenoxyphenyl)acrylamido)phenyl 3-(3-phenoxyphenyl)acrylate* (**2g**). Yield: 35%; R_f_ (toluene/ethyl acetate 2:1): 0.6; m.p.: 181–183 °C; IR (KBr, cm^−1^): 3307.2, 1699.8, 1670.3, 1604.1, 1558.2; LC-MS (*m/z*): (C_36_H_27_NO_5_) [M]^+^ = 553, [M+1]^+^ = 554, [M−1]^+^ = 552, [M+Na]^+^ = 576, [M+K]^+^ = 592, [M+Na+HCOOH]^+^ = 598; ^1^H-NMR (CDCl_3_, δ): 6.47–6.42 (d, 1H, *J =* 15 Hz), 6.52–6.57 (d, 1H, *J =* 15 Hz), 6.98–7.11 (m, 6H, aromatic), 7.14–7.18 (m, 4H, aromatic), 7.31–7.38 (m, 8H, aromatic), 7.55 (s, 1H), 7.65–7.70 (d, 1H, *J =* 15 Hz), 7.77–7.82 (d, 1H, *J =* 15 Hz); Analysis C, H, N. Expected %: (C_36_H_27_NO_5_) C: 78.1, H: 4.92, N: 2.53; Found %: C: 77.8, H: 5.26, N: 2.68.

#### 3.2.5. Synthesis of Hybrid Cinnamic Amide **2h**

Benzocaine (0.8 mmol) was dissolved in pyridine (1.7 mL) and added to a solution of the cinnamoyl chloride of acid **2** (0.8 mmol) in dichloromethane (50 mL). The reaction mixture was refluxed and after 4 h (monitored by TLC) dichloromethane was removed under reduced pressure. The residue was received with ethyl acetate, washed with water and dried over MgSO_4_. The hybrid cinnamic amide **7** was purified chromatographically (PLC, ethyl acetate/petroleum ether 1:3).

*(E)-Ethyl 4-(3-(3-phenoxyphenyl)acrylamido)benzoate* (**2h**). Yield: 25%; R_f_ (petroleum ether/ethyl acetate 3:1): 0.7; m.p.: 210–214 °C; LC-MS (*m/z*) (C_24_H_21_NO_4_) [M+H]^+^ = 388, [M−H]^+^ = 386, [M+Na]^+^ = 410, [M+K]^+^ = 426, [M+Na+MeOH]^+^ = 442; ^1^H-NMR (CDCl_3_, δ): 1.34 (t, 3H, *J =* 9 Hz, **-**CH_3_), 4.32 (q, 2H, *J =* 9 Hz), 6.38 (d, 1H, *J =* 15 Hz), 6.58 (d, 1H, *J =* 9 Hz), 6.93–7.02 (m, 3H, aromatic), 7.06–7.14 (m, 2H, aromatic), 7.21–7.33 (m, 4H, aromatic), 7.7 (d, 1H, *J =* 6 Hz), 7.68 (d, 1H, *J =* 15 Hz), 7.84 (d, 1H, *J =* 9 Hz), 7.98 (d, 1H, *J =* 6 Hz), 8.53 (s, 1H, -NH-C=O); Analysis C, H, N. Expected %: (C_25_H_21_NO_4_ ) C: 74.4, H: 5.46, N: 3.62; Found %: C: 74.77, H: 5.82, N: 3.64.

### 3.3. Physicochemical Studies

#### Determination of R_M_ Values

Reversed phase TLC (RP-TLC) was performed on silica gel plates impregnated with 5% (v/v) liquid paraffin in light petroleum ether. The mobile phase was a methanol/water mixture (70/30, v/v). The plates were developed in closed chromatography tanks saturated with the mobile phase at 24 °C. Spots were detected under UV light. R_M_ values were determined from the corresponding R_f_ values (from five individual measurements) using the equation R_M_ = log [(1/R_f_) − 1] (Table 1) [10,55].

### 3.4. Biological in Vitro Assays

A stock solution of the tested compounds in DMSO (less than 1% in final concentration) was used. Each *in vitro* experiment was performed at least in triplicate and the standard deviation of absorbance was less than 10% of the mean.

#### 3.4.1. TLC Screening for Free Radical Scavengers

TLC was used for screening the synthesized hybrids as radical scavengers. The compound solutions in DMSO were spotted onto a silica gel F_254_ precoated plate and developed using 0.5% MeOH and 95% CHCl_3_ as developing solvents. After development the plates were air-dried and sprayed with 0.1 mM DPPH ethanolic solution for detecting the compounds with rapid scavenging properties. The compounds that show white spots on a purple background most rapidly were considered as active.

#### 3.4.2. Inhibition of Linoleic Acid Lipid Peroxidation

Production of conjugated diene hydroperoxide by oxidation of sodium linoleate in an aqueous dispersion is monitored at 234 nm. AAPH is used as a free radical initiator [12,24]. Ten microlitres of the 16 mM sodium linoleate solution was added to the UV cuvette containing 930 μL of 0.05 M phosphate buffer, pH 7.4 prethermostated at 37 °C. The oxidation reaction was initiated at 37 °C under air by the addition of 40 mM AAPH solution (50 μL). Ten μL of the tested compounds were added in the mixture. Lipid oxidation was measured in the presence of the same level of DMSO. The rate of oxidation at 37 °C was monitored by recording the increase in absorption at 234 nm caused by conjugated diene hydroperoxides. The results were compared to the appropriate standard inhibitor trolox (63%) (Table 1).

#### 3.4.3. Soybean Lipoxygenase Inhibition Study *in Vitro*

DMSO solution of the tested compound was incubated with sodium linoleate (0.1 mM) and 0.2 mL of soybean lipoxygenase solution (1/9 × 10^−4^ w/v in saline) in buffer pH 9 (tris) and at room temperature. [11]The conversion of sodium linoleate to 13-hydroperoxylinoleic acid was recorded at 234 nm and compared with the standard inhibitor NDGA (IC_50_ = 51.5 μM). The results are given in Table 1 expressed as IC_50_ values or % inhibition at 100 μM.

#### 3.4.4. Inhibition of Trypsin Induced Proteolysis *in Vitro*

The test was carried out according to a modified Kunitz method [56]. Bovine albumin was used as substrate for trypsin. The reaction mixture consisted of 0.075 mg/mL trypsin (200 μL) and 780 μL of phosphate buffer pH 7.6 (including the tested compounds (20 μL in DMSO, final concentration 100 μM) preincubated at 37 °C for 20 min and then 1 mL of albumin (stock solution 6 g/100 mL in phosphate buffer 0.1 M, pH 7.6) was added and incubated at 37 °C for 30 min. After the incubation 1 mL of 5% trichloroacetic acid was added to the incubated solution to stop the enzyme’s reaction and allowed to set at room temperature for 1 h. Then the solution was filtered by filter paper and the absorption of the filtered solution was measured at 280 nm. Salicylic acid was used as a reference compound (Table 1).

#### 3.4.5. *In Vitro* Esterase Activity on Hybrids **2a** and **2b**

Representative hybrid molecules are subjected to an esterase activity *in vitro*. 100 μL of 1 mg/mL 0.001 N hydrochloric acid of esterase solution was added to 1,250 μL of phosphate buffer (0.1 M) pH 7.8 including the tested compounds (150 μL in DMSO, final concentration 1 mM) and incubated at 37 °C for 90 min. The incubated solution was allowed to set at room temperature. Then, the absorption was measured at 256 nm. The measurement was repeated after 24 h of incubation at 37 °C.

#### 3.4.6. *In Vitro* Stability of Hybrid Molecule **2a** under Acidic and Basic Conditions

In an attempt to mimic the acidic/basic conditions in the gastrointestinal route, measurements at pH: (i) 3; (ii) 4.5 and (iii) 9 were performed. The pH value was adjusted with the appropriate buffer (CH_3_COONa/CH_3_COOH) to 4.5 and 3 and Tris to pH 9. 20 μL of the tested compounds in DMSO and buffer 980 μL (4.5 or 3 pH) incubated at 37 °C for 24 h. The stability of the compounds was monitored chromatographically after 3 and 9 h. After 24 h, the incubated solution (in all cases) was treated with chloroform (1 mL). The organic layer was analyzed spectrophotometrically.

### 3.5. In Vivo Assays

Anti-inflammatory tests were conducted on Fisher-344 inbred rats of *ca*. 200 g body weight whereas antinociception and motor and sensory functions after sciatic nerve crash were performed on Wistar rats. Both sexes were used. Females pregnant were excluded. Each group was composed of 6 animals. The animals, which have been bred in our laboratory, were housed under standard conditions and received a diet of commercial food pellets and water *ad libitum* during the maintenance. Our studies were carried out in accordance with recognised guidelines on animal experimentation.

The tested compounds 0.01 mmol/kg body weight were suspended in water with few drops of Tween 80 and ground in a mortar before use. Controls received the liquid vehicle only. All the *in vivo* experiments were repeated at least in triplicate and were subjected to statistic analysis using the student’s *t*-test.

#### 3.5.1. Antinociceptive Screen

The writhing reflex was used [57]. The test compounds were administered *i.p*. 30 min prior to the i.p. administration of 1 mL/100 g body weight of 0.6% acetic acid. Paracetamol at 0.1 mmol/100 g body weight was administered *i.p*. as a standard drug for comparison. After 5 min, the number of stretches characterized by repeated contractions of the abdominal musculature accompanied by extension of the hind limbs was counted every 5 min for the next 30 min. The total number of writhes exhibited by each animal in the test group was recorded and compared to that of a vehicle treated control group. The % antinociceptive activity (a:a) was calculated as: % a:a = (n − n'/n) × 100 (a: a analgesic activity, n average number of writhes of control group, n' average number in the test group). For the tested compounds the analgesic activity is shown in Table 1 (Table 1; Figure 1).

#### 3.5.2. Inhibition of the Carrageenin-Induced Edema

Oedema was induced in the right hind paw of Fisher 344 rats by the intradermal injection of 0.1 mL 2% carrageenin in water. Each group was composed of 6 animals which they were entirely fasted during the experiment period. The tested compounds 0.01 mmol/kg body weight, were given intraperitoneally simultaneously with the carrageenin injection. The rats were euthanized 3.5 h after carrageenin injection. The difference between the weight of the injected and not-injected paws was calculated for each animal [10]. The change in paw weight was compared with that in control animals (treated with water) and expressed as a percent inhibition of the oedema (ICPE % values in Table 1). Indomethacin 0.01 mmol/kg was used as reference compound. ICPE % are the mean values from two different experiments with a standard error of the mean less than 10%.

#### 3.5.3. Induction of Sciatic Nerve Injury

One day prior to the surgery and the behavioral testing, Wistar rats were handled by the investigators and introduced to the testing environment for a brief period of time. Rats were anesthetized with chloral hydrate 4.5% (1 mL/100 g). The sciatic nerve of the left hind paw was revealed after blunt dissection through biceps femoris and freed from the surrounding connective tissue. After the exposure, the nerve was crushed once for 30 s at standardized force. The muscle was reapproximated and the skin incision was closed using 6 to 0 sutures. The examination of the loss of sensory and motor function in the operated limb established the crush completeness. Digits in the operated limb were pinched with a blunt forceps. Absence of foot withdrawal and vocalization was recorded as loss of sensory and motor function. Animals were placed under a heating lamp until they recovered from the anesthesia and they returned to their home cages.

#### 3.5.4. Drug and Time of Administration

The control group did not receive any drug after the surgery. A single dose of paracetamol (0.01 mmol/mL/kg) and of hybrid **2a** was administered to the treated animals intraperitoneally, 8 h after the surgery. The tested solutions were freshly prepared on surgery day (day 0).

#### 3.5.5. Locomotor Testing

The animals were subjected to a locomotor testing one day before the surgery and on the 1st, 8th, 15th and 22nd day after the sciatic nerve injury. The day of the surgery is considered as day 0 [36,37,38].

#### 3.5.6. Flat Runway

Rats were trained to walk on a wooden board (1 m × 15 cm) for 120 s. The hindlimb movement was assessed with the scoring mentioned before. The results are given in Figure 3.

#### 3.5.7. Inclined Plane Runway

In this test a wooden board (1 m × 15 cm) fitted at a 45° angle to the ground was used. Rats were trained to walk over the runway for 120 s and their ability to balance their body position was evaluated with the above mentioned scoring. The results are given in Figure 4.

#### 3.5.8. Grid Runway

A wooden apparatus (50 cm × 40 cm × 40 cm) was used in this test. A metallic horizontal grid with square holes (3 cm × 3 cm) was placed 10 cm above apparatus’s base. The roof of the apparatus was open. The 50 cm side was also open from the base until the grid, so that the lower surface of the grid was visible to the investigators. The animals were evaluated as they were walking on the grid and the number of errors (hindlimb slips) was determined. The results are given in Figure 5.

#### 3.5.9. Sciatic Functional Index (SFI)

In the present study, the plantar surfaces of the hindlimbs of the rats were impregnated with ink and then the animals were allowed to walk on a paper along a corridor, leaving footprints during walking [32,58].

A wooden corridor (1 m × 8 cm) with raised sidewalls was used. Two and a half white paper strips (40 × 7.5 cm) were placed along the corridor and were fixed with adhesive tape closely together and at the base and the sidewalls of the corridor. Each rat was trained to walk steadily along the corridor without stops until it made three at least successful efforts of continuous walking. The footprints were allowed to dry and were then converted into digital images using a computer and a high resolution scanner. The image processing was done by using the ImageJ. This is an open-source image analysis software created in 1997 by Rayne Rasband (National Institute of Health), widely used in medical applications, especially neuroscience, for measuring distances and surface and plotting the data [59]. The footprints were analyzed as described by Bain *et al.* [60] in measurements were included:

(i) print length (PL): distance from the heel to the third toe,
(ii) toe spread (TS) distance from the first to the fifth toe on both sides and
(iii) intermediate toe spread (ITS) the distance between the middle of the second and fourth toe

All measurements were taken both from the experimental (EPL, ETS, EIT) and the normal (NPL, NTS, NIT) side. The formula used to calculate SFI was the following:

SFI = −38.3 (EPL − NPL)/NPL + 109.5 (ETS − NTS)/NTS + 13.3 (EIT − NIT)/NIT − 8.8

#### 3.5.10. Sensory Testing Pinch Test

The lateral side of the rat’s paw was pinched with a clip. Animals demonstrating a withdrawal response to pinching were recorded. Painful stimuli should be applied on the outer lateral side of the plantar surface, since the medial side of the sensory function is originated from the saphenous nerve. The tests were performed one day before the operation, on the day following the operation and were repeated at 1-week intervals for three weeks postoperatively. The pinch tests were evaluated with the previous modified score technique (Table 3). The results of the above assay are given in Figure 7.

## 4. Conclusions

The present study shows that the synthesized cinnamic acid hybrids represent a promising class of multitarget compounds influencing several biological targets, e.g., lipoxygenase and trypsin. The hybrid **2a** of phenyloxyphenyl acid with paracetamol showed high analgesic activity (91%) and it appears to be a promising agent for treating peripheral nerve injuries. The esters were found very potent and especially the derived from paracetamol and *m*-acetamidophenol. The amides follow. The hybrids from acid **II** were also found to be more potent than those derived from the other cinnamic acids. Allosteric interactions might govern the LOX-inhibitor binding. Compounds **2a** and **2e** presented a promising combination of *in vitro* activities and high anti-nociception response. Hybrid **3b** presented also an interesting biological profile *in vitro*. Thus, the designed hybrids succeeded to be used as new multifunction agents.

## Abbreviation

AAPH: 2,2-azobis(2-amidinopropane) dihydrochloride
ABTS: 2,2' azinobis(3-ethylbenzothiazoline-6-sulfonic acid)
ACPYPE: AnteChamber PYthon Parser interface
BOP: *O*-(benzotriazol-1-yl)-*N*,*N*,*N*',*N*''-tetramethyluronium hexafluorophosphate
clog *P*: Theoretically calculated lipophilicity
DPPH: 1,1-diphenyl-2-picrylhydrazyl radical
ET: electron transfer
HAT: hydrogen atom transfer
4-HC: 4-hydroxycoumarin
7-HC: 7-hydroxycoumarin
LOX: Lipoxygenase
NDGA: nordihydroguaiaretic acid
m-AP: m-aminophenol
p-AP: p-aminophenol
RPTLC: Reverse-phase thin layer chromatography
PA: paracetamol
